# A best-worst scaling survey of medical students’ perspective on implementing shared decision-making in China

**DOI:** 10.1186/s12909-020-02406-9

**Published:** 2020-12-02

**Authors:** Richard Huan XU, Lingming ZHOU, Eliza Lai-Yi WONG, Dong WANG, Guo Chun XIANG, Chao XU

**Affiliations:** 1 JC School of Public Health and Primary Care, the Chinese University of Hong Kong, Hong Kong SAR, China; 2 School of Health Management, Southern Medical University, Guangzhou, China; 3 Graduate School, Southern Medical University, Guangzhou, China

**Keywords:** Best-worst scaling, Medical students, Shared decision-making, Preference heterogeneity, China

## Abstract

**Background:**

The objective of this study was to ascertain the importance rankings of factors affecting the implementation of shared decision-making (SDM) in medical students in China and determine whether these factors were consistent across the respondents’ individual characteristics.

**Method:**

Students studying clinical medicine were recruited from three medical universities in China. A cross-sectional online survey using best-worst object scaling with a balanced incomplete block design was adopted to investigate their preference towards implementing SDM in China. Count analysis, multinomial logit analysis and mixed logit analysis were used to estimate the preference heterogeneity of the SDM factors among respondents.

**Results:**

A total of 574 medical students completed the online survey. The three most important factors for implementing SDM were trust and respect, (providing) high-quality medical information and multi-disciplinary collaboration. The mixed logit regression model identified significant heterogeneity in SDM preferences among respondents, and sub-group analysis showed that some heterogeneities varied in respondents by sex, study programs and their experience of visiting doctors.

**Conclusion:**

The importance rankings provide rich information for implementing SDM and facilitate the reform of education in medical schools in China. However, the heterogeneities in SDM preference need further explorations.

**Supplementary Information:**

The online version contains supplementary material available at 10.1186/s12909-020-02406-9.

## Background

Shared decision-making (SDM) is a process in which a patient and her/his healthcare professionals work together as decision-making partners. It offers a structured way to incorporate evidence as well as patient values and preferences into medical decision making [1]. SDM helps patients to understand what kind of health care is important to them, and feel supported and empowered to make choices about their health care. It also helps health and social care professionals to tailor the care to meet the needs of individual patients. Ultimately, it is an efficient way to improve patient health outcomes [2]. Although, currently, a growing number of policymakers and medical professionals have encouraged the normalizations of principles of SDM in clinical practice during the past four decades [3–5], the adoption of SDM into clinical practice has been remarkably slow [6].

Several studies have indicated that SDM could bring some medical benefits, such as improved adherence to doctor’s instructions, stronger patient functioning, controlled expenditures and improved health outcomes [7–9]. To the best of our knowledge, currently, some key factors challenge the ability to incorporate SDM into routine practice. First, healthcare professionals might not understand what SDM is in their usual practice. Many doctors believe that they already engage their patients in the decision-making process, although the patients are not participating in the process [10, 11]. Second, healthcare professionals might not know how to include SDM in their usual practice because there are no tools to help them or to facilitate SDM [6]. Third, healthcare professionals might have difficulty understanding patients’ willingness to be involved in SDM or how to determine what types of decisions to share with their patients [12, 13]. One study found that patients who were partly involved in the decision-making process had a higher quality of life than those who were fully or not at all involved in the process [14]. Fourth, the components or features necessary to assess the effectiveness of SDM to promote usage are not yet clear [15]. To be sure, these challenges pose some uncertainties for the healthcare system along the lines of whether healthcare professionals, particularly doctors, are prepared to create a new type of relationships with their patients [9].

Recently, in China, there are no official policies for fostering the cultural change among doctors that is needed to implement SDM as a routine practice [16]. Although the doctor-patient relationship is not as imbalanced as it was the recent past, this relationship’s power differential persists. Because the shift away from the paternalistic medical culture to SDM is a challenging task, there is a consensus that SDM should be introduced as early as possible during medical education and training, which is when medical students have space and time to develop SDM knowledge and skills [17, 18]. However, little evidence exists to inform what strategies may be most effective for normalizing SDM for medical school students [19]. In China, medical training in SDM is underdeveloped, despite the increasing attention being paid to it, and the important factors for facilitating or encouraging medical students to implement SDM in their future medical practices are ambiguous. It is important to understand their attitudes towards SDM during the formative stage of their training before professional behaviours and perspectives are established. Thus, this study aimed to identify ranked importance among factors related to implement SDM in clinical practice from the medical student perspective using the best-worst scaling (BWS) method.

## Method

### Study design and context

The basic system of medical education in China has two tiers. The first is undergraduate education. It is normally a five-year study, including a one-year internship (clinical rotation), after than, the Medical Bachelor degree is awarded. The second is clinical specialization (postgraduate study). It usually involves a three-year Master degree and/or another three-year Doctoral degree study. All the graduates with the degrees of Bachelor, Master or Doctor could apply to be a registered medical practitioner after pass the national examination. The details of the system of medical education in China could be found anywhere [20, 21].

Currently, in China, the curriculum about the concept and application of SDM is not a compulsory module in the system of medical education. Only students who studying clinical medicine, which means they are eligible to register as a doctor in the future career, may select one to two courses about SDM at the last one to two years of their undergraduate study or the first year of the postgraduate study. Thus, in this study, an online cross-sectional BWS survey using a sample of students, who studying clinical medicine, from three medical universities in Guangdong, was conducted.

### Best-worst scaling method

BWS is a method used to measure individual preferences. It has increasingly been employed in healthcare studies in the past few years [22]. BWS has been categorized into three types: (1) Case 1 (object case), Case 2 (profile case), and Case 3 (multi-profile case). In Case 1, researchers explore a population of interest’s preferences regarding each item of a particular list of objects. The individuals under observation are presented with a set of objects and asked to separately choose the one best and the one worst object. In Case 2, researchers focus on the importance ranking of items presented in a single profile structure presented to the individuals, which is developed by combining attributes at various levels. In Case 2 designs, the individuals are not asked to compare the benefits among various profiles, but instead to choose one attribute level combination as the best one and another attribute level combination as the worst one in that particular profile, e.g. Ratcliffe et al.’s study [23]. Case 3 presents multiple profiles to the individuals from which to choose the best one and the worst one from each choice set, e.g. Thong et al.’s study [24]. The theoretical, methodological, and analytical details can be found in Finn and Louviere [25]. This study employed the BWS Case 1 method.

### Generation of BWS factors

In order to generate the SDM-related factors to be used in the BWS survey, four steps were conducted. The first step was an extensive literature review. Two teams of researchers carried out the literature review, respectively. Medline, Embase, Web of Science, and PsycINFO were used to search the relevant papers. Studies were included if they focused on both perceived barriers and facilitators to implementing SDM in practice and medical education, involved the conceptualizations, the structures, and instruments to assess the SDM, from the perspectives of both health professional and patient All the findings were synthesized and jointly discussed by two research teams. At the end of first stage, 19 SDM-related factors were found and summarized for the following discussions (the list of factors is presented in Table S1, supplementary file). The second step was a focus group interview. Fifteen focus groups, including five doctor, nurse, and patient groups, respectively, in five cities (Guangzhou, Shenzhen, Meizhou, Zhanjiang, and Shaoguan) were carried out. The aim was to further discuss the concepts of SDM and the barriers and facilitators of its implementation in daily practice. Semi-structured interviews were used and, consequently, 15 factors were summarized for further refinement. The third step was expert discussion. Findings from the literature review and the focus groups were summarized by the research team and presented to a multi-disciplinary expert group for final discussion and confirmation. Four experts with rich experience in SDM research were invited, including two doctors and two researchers. After two rounds of face-to-face discussion, a draft of 13 SDM-related factors was confirmed. In order to assess the validity of the SDM-related factors, 10 medical students and 10 healthcare professionals (five doctors and five nurses) were invited to participate in a pilot survey. Respondents were asked to rank the five most important factors and provide reasons using their own language. An open-ended question was also settled at the end of the survey to prompt comments about the SDM-related concepts or factors. After revisions, a list of 13 SDM-related factors was finally developed (Table 1).

**Table 1:** List of factors and Description

	Factors^#^	Abbreviation	Descriptions
1	Multi-disciplinary collaboration	MC	Strengthen the collaboration of different disciplines in order to help patients to know their health problems from different perspectives
2	Patient collaboration	PC	Improving and encouraging the collaborations between patients
3	(Providing) High-quality medical information	MI	Providing a variety of channels to help patients to gain the knowledge and skills they need
4	(Building) Trust and respect	TR	Building trusted and respected professional-patient relationship
5	(Providing) Health education	HE	Providing health education for patients to improve their ability for self-care
6	Provision of decision aids tools	DA	Providing the best available evidence to facilitate doctors to make clinical decisions
7	(Controlling) Number of patients	NP	Control the number of patients a doctor meet and treat every day
8	(Providing) Administrative support	AS	The managers and leaders provide supports for improving SDM at political level in the hospital.
9	Assistance of Family/ caregivers	FA	The family members or caregivers actively join in the health care
10	Financial incentives	FI	Providing financial motivation for encouraging doctors to engage SDM
11	(Protecting) Privacy in Clinics	CP	Protecting patient’s privacy and make them feel comfort to communicate with doctors
12	(Improving) Communication skills	CS	Educating and improving the doctors’ communicating skills to improve SDM
13	(Providing) SDM training	ST	Providing SDM trainings for health professionals to help them understand what it is and how to do it

# All the factors and explanations were presented in Simplified Chinese during the online survey

### The design of the BWS questionnaire

**Fig. 1:**
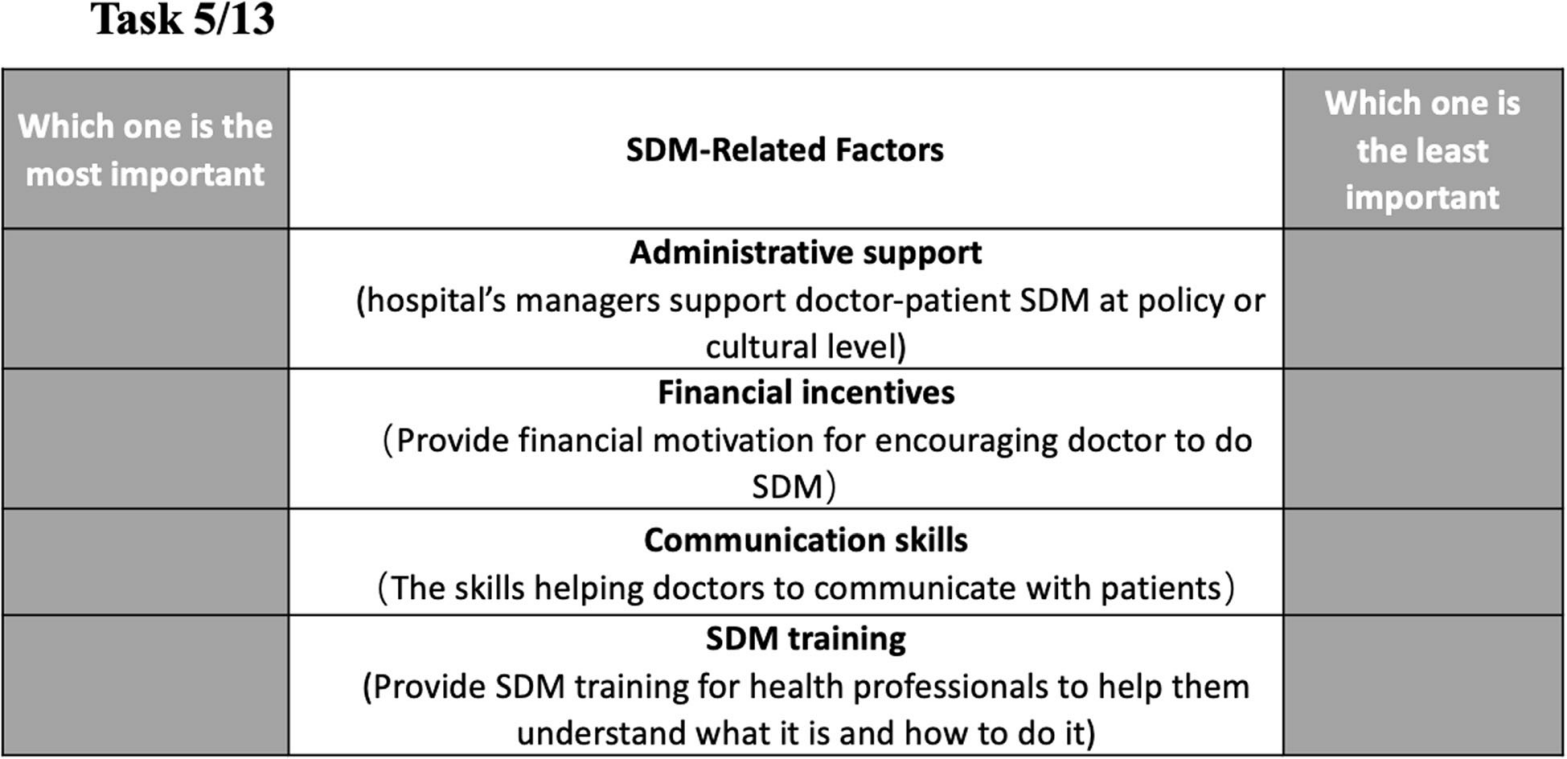
Example of the choice task

A balanced incomplete block design (BIBD), the most commonly used designs by far for BWS object cases, was used to construct our choice tasks [26]. BIBD is the optimal way to create a design in which every attribute level (factor) is equally replicated and appears within the block (choice tasks) with every other attribute level (factor) an equal number of times [27]. The BIBD ensures equal probabilities of selecting factors. The details of the BIBD methodology could be found anywhere [26]. Table 2 shows that, in this study, there was a total of 13 choice tasks, each with four factors. For each choice task, a brief introduction of the study and description of included factors was provided. An example of the choice task is shown in Fig. 1. The respondents were asked to select a most important and a least important factor that could promote the implementation of SDM in the clinical practice from their perspective. All the respondents had to complete 13 choice tasks in total. Overall, 26 choices were made by each respondent.

**Table 2:** Experimental design

Factors	Choice tasks												
	CT1	CT2	CT3	CT4	CT5	CT6	CT7	CT8	CT9	CT10	CT11	CT12	CT13
**MC**	0	1	1	0	0	0	0	0	1	0	0	0	1
**PC**	0	0	0	0	0	0	1	1	1	0	0	1	0
**MI**	1	1	0	0	0	0	0	1	0	0	1	0	0
**TR**	0	0	1	1	0	0	0	0	0	0	1	1	0
**HE**	0	0	0	1	0	1	0	1	0	0	0	0	1
**DA**	0	1	0	0	0	1	0	0	0	1	0	1	0
**NP**	0	0	0	0	0	0	1	0	0	1	1	0	1
**AS**	0	0	1	0	1	0	0	1	0	1	0	0	0
**FA**	1	0	1	0	0	1	1	0	0	0	0	0	0
**FI**	0	0	0	0	1	1	0	0	1	0	1	0	0
**CP**	1	0	0	1	0	0	0	0	1	1	0	0	0
**CS**	1	0	0	0	1	0	0	0	0	0	0	1	1
**ST**	0	1	0	1	1	0	1	0	0	0	0	0	0

Currently, the optimal sample size for conducting a BWS study is inconclusive. Lancsar and Louviere proposed that more than 20 respondents per questionnaire version would estimate reliable models [28]. Meanwhile, the latest review reported that sample sizes using in the BWS object case studies ranged from 15 to 803, with a median sample size of 175. Additionally, a trend in decreasing sample size was observed [22]. Therefore, in this study, we chose to obtain a minimum convenience sample of 500 individuals.

### Data collection

With the assistance of the tutors in each university, data were collected though an online surveying platform from June to August in 2019. All the students, both undergraduate and postgraduate, from the targeted universities received a recruitment message that included a brief description of the study’s purpose, the basic concepts and mechanics of BWS and a hyperlink to the online questionnaire. The questionnaire had three parts: an informed consent, questions about the respondents’ characteristics (sex, age, studying programme, degree level, experience of employment and etc.), and the BWS questions. Students who submitted completed questionnaires were entered into a raffle to win a prize (RMB 2.5 on average (about USD 0.15). The institutional review board of the second clinical school of Guangzhou Medical University (No. 2019-ks-08) approved the study protocol and informed consent.

### Statistical analysis

We analysed BWS response data in two ways. Count analysis was conducted to examine the choice frequency. The best-worst scaling score (BW score) was calculated as the number of times a factor was selected as the ‘most’ important minus the number of times that factor was selected as the ‘least’ important for each of the 13 factors. A positive BW score indicated the factor was chosen more often as ‘most’ than as ‘least’ important and a negative BW score indicated the reverse [29]. Scaled BW score was reported based on Uy et al.’s method, that is, it was computed by the square root of the total best score divided by the total worst score for each factor. The scaled BW score indicated the choice probability relative to the most important factor [30]. The mean BW score was also reported, which was calculated by the total BW score divided by the number of respondents who responded to that factor. All the BWS questions were pre-defined as compulsory in the online survey, thus, no missing data needed to be imputed in our analysis.

Multinomial logit analysis (MNL) and mixed logit model (MXL), which incorporate the logit procedure (where each factor has dual coding, best = 1 if the factor is chosen as most important, and best = 0 if otherwise; meanwhile, and worst = 1 if the factor is chosen as least important, and worst = 0 if otherwise), were used to estimate propensity scores capturing the probability that a factor was present in a specific combination of factors [31]. The equation presents the relationship between the difference in utility between the best and worst (${U}_{diff}^i$ on the latent utility scale) for choice task *i* (*i* = 1, 2, 3 … 13), and the 13 independent variables (factors). The standard MNL and MXL function links the observed discrete choice (0 or 1) with the estimated latent utility [32].

$$ {U}_{diff}^i={\beta}_{MC}{D}_{MC}^i+{\beta}_{PC}{D}_{PC}^i+{\beta}_{MI}{D}_{MI}^i+{\beta}_{TR}{D}_{TR}^i+ $$

$$ {\beta}_{HE}{D}_{HE}^i+{\beta}_{DA}{D}_{DA}^i+{\beta}_{NP}{D}_{NP}^i+{\beta}_{AS}{D}_{AS}^i+ $$

$$ {\beta}_{AF}{D}_{AF}^i+{\beta}_{FI}{D}_{FI}^i+{\beta}_{CP}{D}_{CP}^i+{\beta}_{CS}{D}_{CS}^i+{\beta}_{ST}{D}_{ST}^i+{\varepsilon}^i $$

For MNL analysis, a coefficient was calculated on every factor reflecting the relative preference of that factor compared to other factors [31]. Odds ratios were also provided to facilitate the explanations of the importance of SDM-related factors. MXL was used to assess the preference heterogeneity. The MXL assumed that the parameters varied across individuals, and, therefore, accounted for the sample’s heterogeneity [28]. Along with the coefficients, the MXL model generated a standard deviation statistic on each factor to indicate the unexplained variation around the mean. Standard deviation terms significantly different from zero indicated significant heterogeneity. One-way analysis of variance (ANOVA) was used to conduct a heterogeneity analysis to assess the difference of BWS scores between different subgroups of respondents (three analyses were conducted on the basis of respondents’ gender [male vs. female], degree level [undergraduate vs. postgraduate] and experience as a patient [with vs. without]). The R statistical program (R foundation, Austria) was used to design the BWS survey and perform all of the statistical tests. Statistical significance was determined by *p*-values ≤0.05.

## Results

### Respondents’ characteristics

The data of 574/1103 students, who completed the online survey and self-reported studying clinical medicine, were elicited for analyses. The respondents were from 27/34 provinces of China, with mean age of 24.8 years, and 61% were female. More than 60% of the respondents were postgraduate students. The most common programme of study were internal medicine (36.9%), surgery and subspecialties (25.4%), and paediatric medicine (12.9%). The average length of work experience was 0.32 years, and average internship was 5.93 months (Table 3).

**Table 3:** Respondents’ characteristics (*n* = 574)

	n	%
**Sex**		
Male	224	39.0
Female	350	61.0
**Degree level**		
Undergraduate	223	38.9
Postgraduate	351	61.1
**Study programme**		
Internal medicine	212	36.9
Obstetrics and gynaecology	53	9.2
Emergency medicine	41	7.1
Paediatrics	74	12.9
Surgery and sub-specialities	146	25.4
Otorhinolaryngology	48	8.4
**Age (**mean, standard deviation [sd]**)**	24.8	3.67
**Working experience (**years [mean, sd]**)**	0.32	1.25
**Internship experience (**months [mean, sd]**)**	5.93	0.72

### Results of BWS survey

In Table 4, ‘trust & respect’ was the most important SDM factor (TR: Factor #4, mean BW score = 1.728). The other factors were ‘High-quality Medical Information’ (MI: Factor #3, mean BW score = 1.247); ‘Multi-disciplinary Collaboration’ (MC: Factor #1, mean BW score = 0.918); ‘Assistance of Family/caregiver’ (FA: Factor #9, mean BW score = 0.122) and ‘Health Education’ (HE: Factor #5, mean BW score = 0.022). The ‘Privacy in Clinics’ factor (CP: Factor #11) was consistently listed as the least important SDM factor (mean BW score = − 1.121).

**Table 4:** Summary of BWS results

	Count analysis					MNL			MXL		
Factors	BW score	Mean BW score	sd of mean BW score	Scaled BW score	sd of scaled BW score						
						b	se	OR	b	se	sd
TR	992	1.728	0.432	2.211	1.000	1.464***	0.045	4.324	1.887***	0.094	1.195***
MI	716	1.247	0.312	1.812	0.821	1.204***	0.044	3.333	1.350***	0.082	0.684***
MC	527	0.918	0.229	1.578	0.713	1.030***	0.043	2.801	0.905***	0.080	0.051
FA	70	0.122	0.030	1.071	0.484	0.619***	0.042	1.858	0.807***	0.056	0.658***
HE	13	0.022	0.005	1.013	0.458	0.589***	0.042	1.802	0.700***	0.066	0.282*
NP	−23	−0.040	− 0.010	0.981	0.444	0.536***	0.042	1.710	0.809***	0.066	0.931***
CS	−43	−0.074	− 0.018	0.954	0.431	0.529***	0.042	1.697	0.617***	0.052	0.236
PC	−65	−0.113	− 0.028	0.953	0.431	0.514***	0.042	1.673	0.499***	0.080	0.978***
DA	− 120	−0.209	− 0.005	0.891	0.403	0.457***	0.042	1.579	0.498***	0.060	0.046
FI	− 420	− 0.732	− 0.183	0.678	0.306	0.201***	0.042	1.222	0.231***	0.054	0.803***
AS	− 440	−0.766	− 0.191	0.671	0.304	0.186***	0.042	1.204	0.158***	0.052	0.106
ST	− 563	−0.981	− 0.245	0.513	0.232	0.073*	0.042	1.076	0.009*	0.055	0.256
CP	−644	−1.121	−0.280	0.461	0.208	Ref	–	–	Ref	–	–

*MNL* Multinomial logit model, *MXL* Mixed logit model, *b* Coefficient, *se* Standard error, *OR* Odds ratio, *sd* Standard deviation;

Reference: CP

* *p* < 0.05; ** *p* < 0.01; *** *p* < 0.001.

Table 4 also reports the relative importance of the SDM factors estimated by the MNL and MXL models. The importance of each factor was estimated relative to CP, which was the least important factor identified by the initial BWS results (see Table 4). Both models found that TR, MI and MC were the three most important SDM factors. FA, HE and ‘number of patients’ (NP) were the next most important factors. All of the factors seemed to be of moderate importance except for ‘SDM training’ (ST), for which the coefficient (coefficient = 0.073/0.009 [MNL/MXL]) was closed to the reference factor (CP). Significant preference heterogeneity was found by the MXL model regarding seven SDM factors (the standard deviations were significantly different from zero). The results, therefore, suggest that the preferences for these SDM factors differed among the grouped respondents. Fig. S1 graphically provides an example of heterogeneity of two factors (‘Patient collaboration’ [PC] and ‘Provision of decision aids tools’ [DA]) with similar mean B-W scores, but different dispersions: PC had almost no variation (standard deviation was non-significant) and DA had wide variation (standard deviation was significant).

### Heterogeneity

**Fig. 2:**
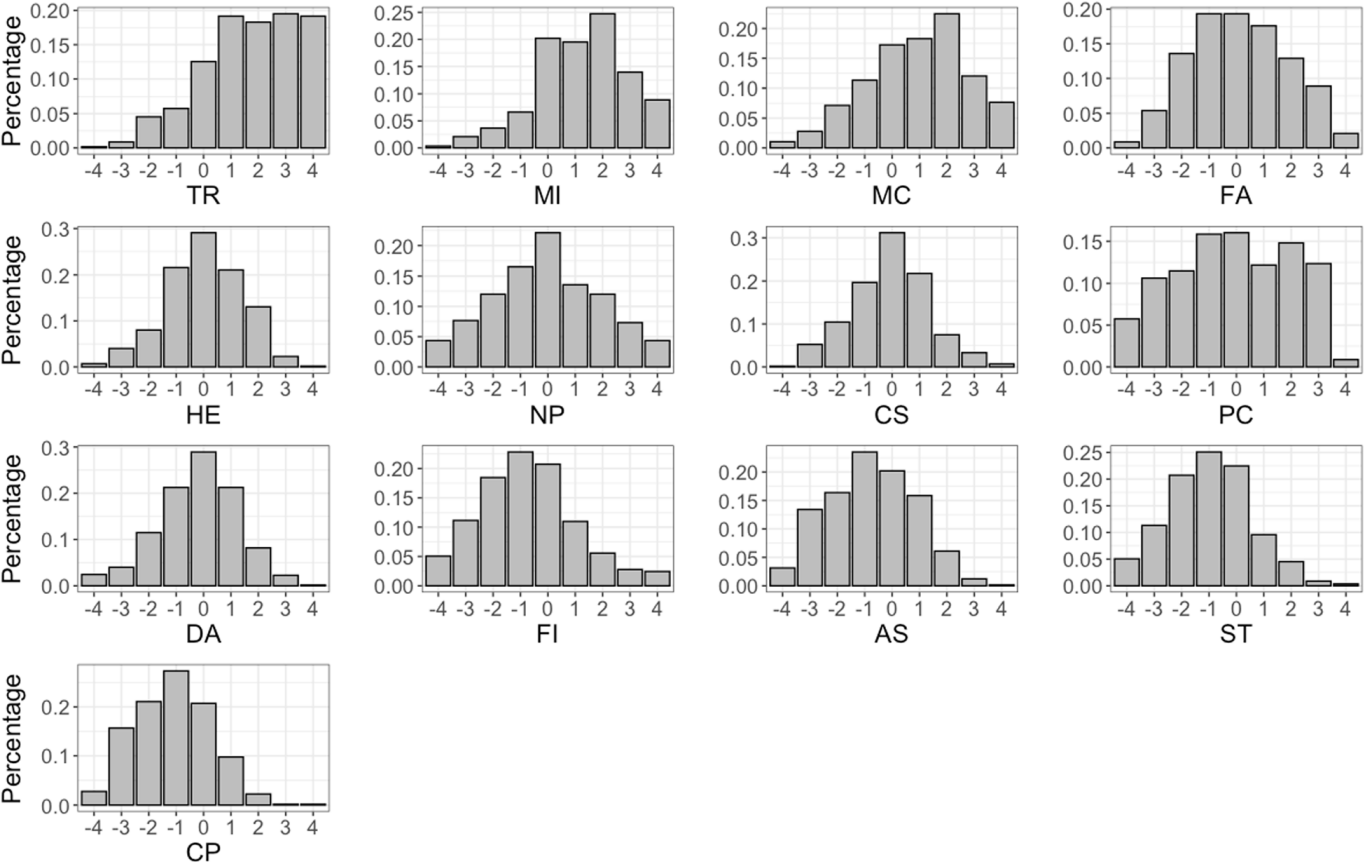
Empirical distribution of individual BW scores

Figure 2 illustrates the distribution of B-W scores of each SDM factor. The distribution indicates that the assumption of normality of the MXL model might not hold. Multi-modal preferences were observed on some factors, suggesting that different types of respondents had different preferences for the factors, and the MXL model’s results should be cautiously interpreted. Table 5 presents the results of the heterogeneity analysis on the subgroups of respondents categorized based on the mean B-W scores. Providing SDM training was more important to the males than to the females, and the females considered protecting patients’ privacy in clinics essential to improving SDM. TR, MC, FA, HE, NP, and ‘communication skills’ (CS) were more important to postgraduate than to undergraduate students. Moreover, the respondents with experience as a patient considered TR, FA, and HE more important for improving SDM than the respondents with no experience as a patient.

**Table 5:** Importance rank of BWS scores by gender, degree level and experience as a patient

Factors	Male vs. female		Undergraduate vs. postgraduate		With experience as patient vs. without	
	Mean in difference	F-value	Mean in difference	F-value	Mean in difference	F-value
TR	−0.06	0.16	−1.21***	71.59	0.64**	15.44
MI	0.09	0.42	0.38*	6.92	−0.13	0.74
MC	0.02	0.01	−0.24*	2.48	−0.08	0.21
FA	−0.15	1.05	−0.80***	28.84	0.46**	7.9
HE	−0.12	0.96	0.39***	10.17	0.35***	7.22
NP	−0.02	0.02	−0.51**	8.84	0.29	2.44
CS	0.19	2.39	−0.62***	26.71	0.22	2.67
PC	0.05	0.09	0.8***	20.3	−0.33	2.83
DA	−0.05	0.13	0.34***	7.48	−0.30	4.8
FI	−0.01	0.01	0.36**	5.78	0.09	0.32
AS	−0.02	0.03	0.47***	11.95	−0.12	0.73
ST	0.36*	7.75	0.05	0.14	−0.13	0.82
CP	−0.28*	5.37	0.59***	25.87	−0.25*	3.89

* *p* < 0.05; ** *p* < 0.01; ***p < 0.001

**Fig. 3:**
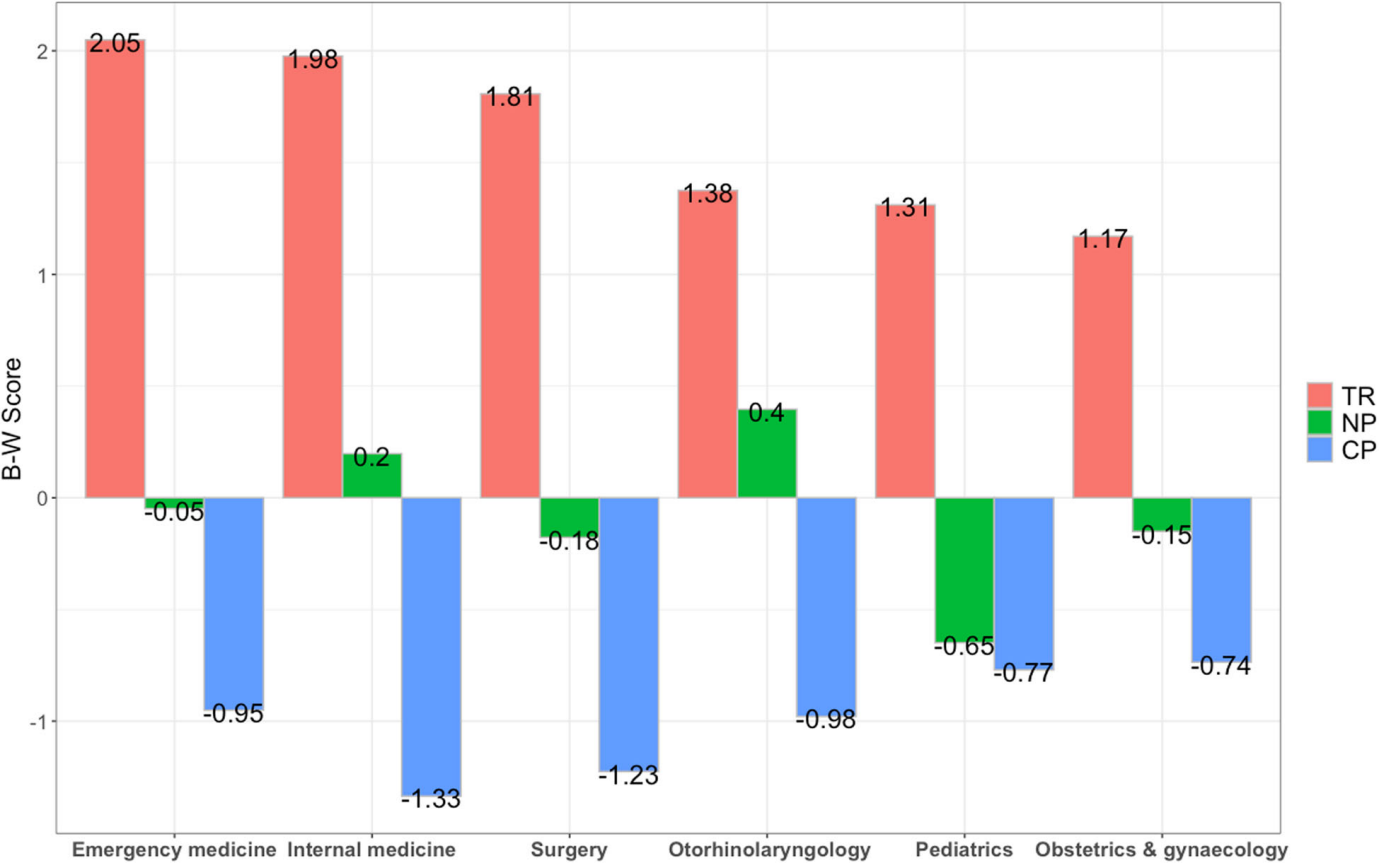
The BW score for respondents with different studying programme on three statistically significant factors

Figure 3 shows that three SDM factors (TR, NP, and CP) had preference heterogeneity among students reported studying different programmes. Respondents studying emergency medicine were most likely to choose TR as the most important factor to improve SDM, followed by respondents studying internal medicine and surgery. Respondents studying otorhinolaryngology indicated that controlling the number of patient visits per day is more important than other factors for improving SDM. CP was the most important factor for respondents studying obstetrics and gynaecology, whereas the respondents studying internal medicine believed it was least important.

## Discussion

Implementing SDM is a challenging, but possible, way to achieve patient-centred care. Our study aimed to determine which of a variety of factors medical students identified were most important for implementing SDM in healthcare practice. We found that trust and respect, high-quality medical information, and multi-disciplinary collaboration were consistently ranked as the three most important factors by medical students. To the best of our knowledge, no studies that have ranked aspects of SDM were found in China, and this study is the first one in China to evaluate the importance of SDM factors using the best-worst scaling method. Notably, this study minimized the variation in scaling and, thereby, provided accurate factor ranking for improving SDM.

Most medical students identified, in this study, that the development of a trustful and respectful relationship between doctors and patients might be the most important factor for implementing SDM in clinical practice, which is consistent with the findings of some previous studies. For example, Kraetschmer et al. demonstrated that patients who preferred shared roles in their healthcare were likely to show moderate to high levels of trust [33]. Meanwhile, a study in the United States (US) found that doctors’ SDM behaviours facilitated patient trust [34]. However, evidence on the relationship between TR and SDM from the medical students’ perspective is very limited. Our study found that a preference heterogeneity existed, with postgraduate students rating it TR less important than undergraduate students. Further evidence is needed to support this conclusion.

Moreover, in our sample, TR was listed as the most important factor among students studying emergency medicine; notably, this was previously observed in a US study that indicated that the creation of an environment of trust, respect, and acceptance is very important in emergency medicine [35]. However, the students studying obstetrics and gynaecology ranked trust and respect as the least important factor; this contradicts the findings of previous studies, which found that respect, dignity, and emotional support were important to women during childbirth [36, 37]. Further research is needed to confirm the consistency of our findings. In addition, our respondents with experience as patients ranked trust and respect higher than respondents without that experience. Osler made the important point that, ‘*in what may be called the natural method of teaching, the student begins with the patient, continues with the patient and ends his study with the patient*’ [38]. Our findings provide empirical evidence that training and encouraging medical students through role-playing as patients may be an efficient way to improve SDM in China [39, 40].

Asymmetry in healthcare information is a long-term problem originating in a medical culture in which the doctor is paternalistic and the patient is passive [10, 12]. In this study, medical students showed providing high-quality medical information to patients is perceived as a key factor to improve the SDM. It is in line with the findings of other studies that delivering high-quality *information* to patients is believed can lead patients and professions to engage in a process of collaboration and deliberation, and make better decisions [41, 42]. Moreover, in compliance with previous evidence, our MXL model identified some variation in perspectives about the importance of MI. Wills et al. found that patients with relatively less education need information than can easily be understood, and that patients with serious health problems tend to be eager for information tailored by doctors [43]. Some other studies indicated that providing information that meets a patient’s needs, preferences and experiences could promote SDM and improve health [42, 44, 45]. Further studies exploring the types of information provided and how such information may help patients to be involved in decision making should be conducted.

Medical students revealed that doctor-patient SDM might be improved through improving their communication skills. Although doctor-patient communications have increasingly gained academic attention [46, 47], the majority of studies have focused on theoretical arguments. In practice, doctors often lack training in communication, which might lead to difficulties responding to patients’ informational and emotional needs [48]. Our study found that the medical students in our sample aware that communicating with patients might be a key component of SDM. However, improving SDM by equipping them with advanced communication skills is a complicated process that requires a cooperative effort with actively engaged patients and a responsive healthcare system [48]. Moreover, we noted that the senior students more strongly supported the importance of communication skills than junior students. In China, medical students have to spend at least one year in internships to fulfil graduation requirements, and the senior students, who are more likely than their less experienced counterparts to have rich experience in clinical practice, might have been realized by the importance of regarding communications in routine practice.

Our results imply that medical students have realized that family members and/or caregivers should not be excluded from the process of decision-making. This finding supports previous studies’ results that involving families and/or caregivers in decision-making is essential for bringing patients into decision-making processes [49]. A US study found that involving family and/or caregivers in decision-making was the key to bringing terminal patients’ values and preferences into treatment decisions [50]. However, currently, in China, most existing studies considered only the involvement of family and/or caregivers in healthcare as it relates to improving doctor-patient communication rather than promoting SDM or patient-centred care.

Degree level (i.e. undergraduate and postgraduate) seemed to be significantly important factor that affect medical students’ attitude toward SDM; notably, similar issues have rarely been discussed worldwide. A recent study found that undergraduate students believed that gaining additional professional skills and receiving organizational support was important to SDM [51]. Another study focused on postgraduate students found that they were more likely than undergraduate students to prefer family involvement in decision-making [52]. Moreover, in this study, the important ranks of SDM factors, such as CP, was also affected by the student’s degree level. However, we need to note that the difference of medical students’ preference toward SDM might be affected by several other factors, such as working setting (outpatient vs. inpatient), patient volume, supervisor’s training style and their knowledge and skills of SDM. Studies are need to further investigate the effect of these factors on medical students’ attitude toward SDM. Our study is a pioneer regarding the importance of SDM; notably, it paints a relatively complex picture of the attitudes and perspectives of medical students. The results imply that medical education should facilitate student ability to improve SDM, with consideration given to student programs and preferences.

Given that no studies using the BWS method have been done in the Chinese healthcare context, our study emerges as an important effort to fill the research gap in methodology. We believe that the BWS method is superior to conventional ranking methods, that is, simple rank ordering or the directly assignment of scores to potential factors, because it strengthens confidence in results by offering benefits such as reduced cognitive burden, easier choice tasks, smaller sample sizes, full rankings as opposed to partial rankings and reducing personal response style bias [30]. BWS, including the Case 2 and Case 3 approaches, is a promising new way to generate rich preference information, and its usage should be encouraged for future studies on China’s healthcare.

Despite its clear contributions, our study has some limitations. First, to control for the influence of the respondents’ cognitive burden, only 13 factors were included and therefore some other important factors related to SDM might not have been included. Second, the BWS Case 1 approach is a parsimonious method for eliciting individual preferences, but it might not provide information as rich as BWS Case 2 or Case 3. Third, although the students in this sample were from 27 of China’s 34 provinces, about one-third of them were Guangdong Province natives. Additionally, around 80 students were first or second year university students, who might have few knowledge about the SDM. Both of them could interfere the ability to generalize our results. Forth, our convenience sample collected through online survey might result in some information and selection bias. Last, we only investigated the medical students’ attitude toward the implementation of SDM, which might be different from the perspective of doctors in clinical practice. It might lead to some problems in generalizability.

## Conclusion

This study used the BWS method to preliminarily explore the importance of factors for implementing SDM from the perspective of Chinese medical students. The study provides rich evidence about the relative importance of the factors at the individual and aggregated levels. The respondents consistently ranked trust and respect, (providing) high-quality medical information and multi-disciplinary collaboration as the most important factors for improving SDM. Future research should investigate the preference heterogeneity regarding SDM among medical students and/or healthcare professionals with a wider range of characteristics.

## Supplementary Information

**Additional file 1.**

## Data Availability

The data that support the findings of this study are available from the corresponding author upon reasonable request.

## Abbreviations

SDM: Shared decision-making
BWS: Best-Worst Scaling
MNL: Multinomial logit regression
MXL: Mixed logit regression
BIBD: Balanced incomplete block design
