# Supplementary material for: A best-worst scaling survey of medical students’ perspective on implementing shared decision-making in China
Source: BMC Med Educ. 2020 Dec 2;20:486. doi: 10.1186/s12909-020-02406-9 (PMC7709333; doi:10.1186/s12909-020-02406-9)
Supplement: Supplementary file 1 — Additional file 1. [file 12909_2020_2406_MOESM1_ESM.docx]

Table S1 the list of SDM-related factors and references

|  | **Factor** | **Ref** |
| --- | --- | --- |
| 1 | Multi-disciplinary collaboration | (Louwers et al., 2018; Parikh et al., 2015; Quinn, Wolczynski, Sroka, &Urman, 2018) |
| 2 | Political support | (Blumenthal-Barby et al., 2019; Levin, Gewirtz, &Cribb, 2017) |
| 3 | Therapeutic techniques | (Crickard, O’Brien, Rapp, &Holmes, 2010; Westermann, Verheij, Winkens, Verhulst, &VanOort, 2013) |
| 4 | Palliative care | (Feuz, 2014) |
| 5 | Health education | (Dubus &Howard, 2016; Simmons, Wolever, Bechard, &Snyderman, 2014) |
| 6 | Clinic environment | (Kelley, Parke, Jokinen, Stones, &Renaud, 2011; M.D., R.C., &V., 2000; Mazurenko &Hearld, 2015) |
| 7 | Transportation | (Chaiyachati et al., 2018) |
| 8 | Privacy | (Sharko, Wilcox, Hong, &Ancker, 2018) |
| 9 | Assistance of Family/ caregivers | (Herrin et al., 2016; Visser, Deliens, &Houttekier, 2014) |
| 10 | Decision aids | (Coulter, Edwards, Elwyn, &Thomson, 2011; van derWeijden et al., 2011) |
| 11 | Financial incentives | (O’Connor, Graham, Visser, A.M., &I.D., 2005) |
| 12 | Social medium | (Diouf, Menear, Robitaille, Painchaud Guérard, &Légaré, 2016) |
| 13 | Patient collaboration | (Blumenthal-Barby et al., 2019; Sharko et al., 2018) |
| 14 | Utilization of Internet technology | (Siegel  Corey, 2007) |
| 15 | Trust and respect | (Kraetschmer, Sharpe, Urowitz, &Deber, 2004; Peek et al., 2013) |
| 16 | High-quality medical information | (Durand, Id, Song, Id, &Id, 2018) |
| 17 | Professional's attitude | (Pollard, Bansback, &Bryan, 2015) |
| 18 | Communication skills | (Feuz, 2014; Kraetschmer et al., 2004; Peek et al., 2013; Sharko et al., 2018) |
| 19 | SDM training | (Diouf et al., 2016; Hauer, Fernandez, Teherani, Boscardin, &Saba, 2011) |


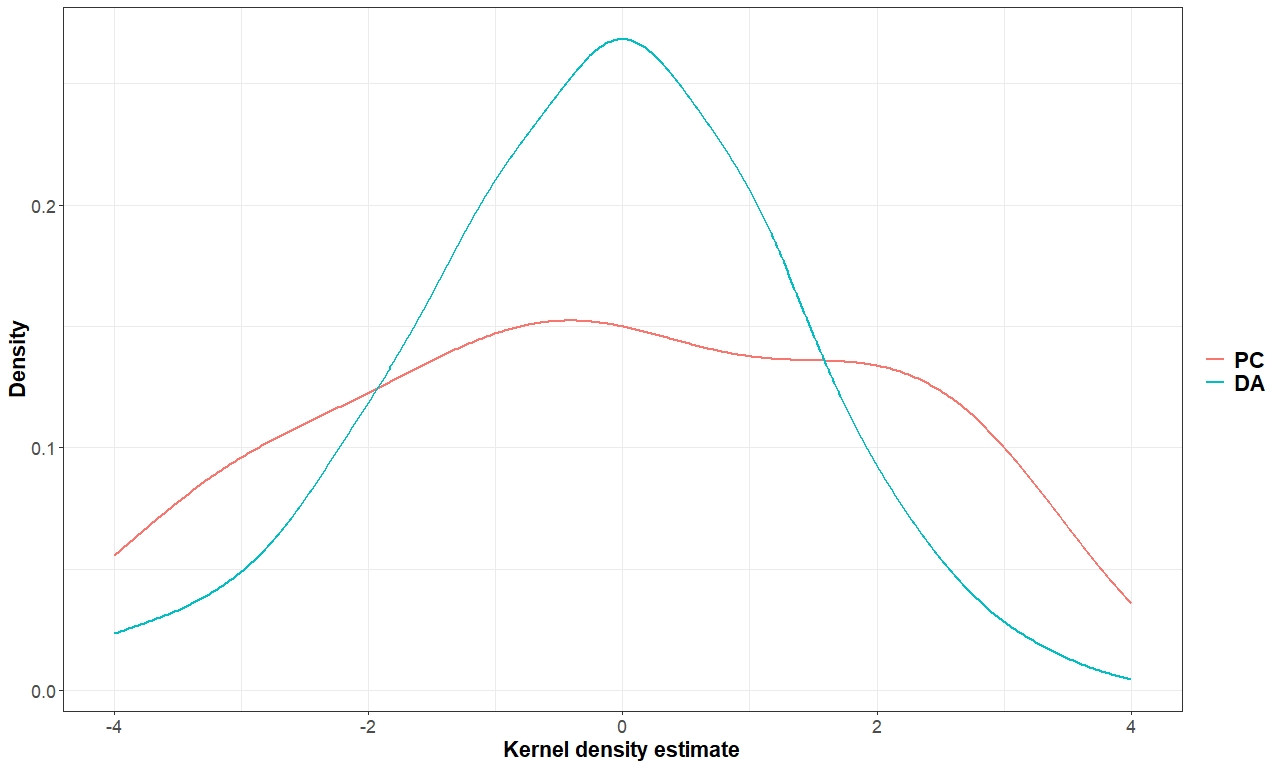


Figure S1 Heterogeneity between item PC and DA

**Reference for Table S1**

Blumenthal-Barby, J., Opel, D. J., Dickert, N. W., Kramer, D. B., Tucker Edmonds, B., Ladin, K., …Tilburt, J. (2019). Potential Unintended Consequences Of Recent Shared Decision Making Policy Initiatives. *Health Affairs (Project Hope)*, *38*(11), 1876–1881. https://doi.org/10.1377/hlthaff.2019.00243

Chaiyachati, K. H., Hubbard, R. A., Yeager, A., Mugo, B., Lopez, S., Asch, E., …Grande, D. (2018). Association of Rideshare-Based Transportation Services and Missed Primary Care Appointments: A Clinical Trial. *JAMA Internal Medicine*, *178*(3), 383. https://doi.org/10.1001/jamainternmed.2017.8336

Coulter, A., Edwards, A., Elwyn, G., &Thomson, R. (2011). Implementing shared decision making in the NHS. *Zeitschrift Fur Evidenz, Fortbildung Und Qualitat Im Gesundheitswesen*, *105*(4), 300–304. https://doi.org/10.1016/j.zefq.2011.04.014

Crickard, E., O’Brien, M., Rapp, C., &Holmes, C. (2010). Developing a Framework to Support Shared Decision Making for Youth Mental Health Medication Treatment. *Community Mental Health Journal*, *46*(5), 474–481. https://doi.org/10.1007/s10597-010-9327-z

Diouf, N. T., Menear, M., Robitaille, H., Painchaud Guérard, G., &Légaré, F. (2016). Training health professionals in shared decision making: Update of an international environmental scan. *Patient Education and Counseling*, *99*(11), 1753–1758. https://doi.org/10.1016/j.pec.2016.06.008

Dubus, N., &Howard, H. (2016). Current realities and future vision: Developing an interprofessional, integrated health care workforce. *Social Work in Health Care*, *55*(9), 766–778. Retrieved from http://ovidsp.ovid.com/ovidweb.cgi?T=JS&PAGE=reference&D=medc&NEWS=N&AN=27649460

Durand, M., Id, P. R. D., Song, J., Id, R. W. Y., &Id, P. J. B. (2018). Shared decision making embedded in the undergraduate medical curriculum : A scoping review. *PLOS ONE*, *13*(11), e0207012.

Feuz, C. (2014). Shared decision making in palliative cancer care: a literature review, *13*(3), 340–349. https://doi.org/10.1017/S1460396913000460

Hauer, K. E., Fernandez, A., Teherani, A., Boscardin, C. K., &Saba, G. W. (2011). Assessment of Medical Students ’ Shared Decision-Making in Standardized Patient Encounters. *Journal of General Internal Medicine*, *4*(4), 367–372. https://doi.org/10.1007/s11606-010-1567-7

Herrin, J., Harris, K. G., Kenward, K., Hines, S., Joshi, M. S., &Frosch, D. L. (2016). Patient and family engagement: a survey of US hospital practices. *BMJ Quality & Safety*, *25*(3), 182–189. https://doi.org/https://dx.doi.org/10.1136/bmjqs-2015-004006

Kelley, M.Lou, Parke, B., Jokinen, N., Stones, M., &Renaud, D. (2011). Senior-friendly emergency department care: an environmental assessment. *Journal of Health Services Research & Policy*, *16*(1), 6–12. https://doi.org/https://dx.doi.org/10.1258/jhsrp.2010.009132

Kraetschmer, N., Sharpe, N., Urowitz, S., &Deber, R. B. (2004). How does trust affect patient preferences for participation in decision‐making? *Health Expectations*, *7*(4), 317–326. https://doi.org/10.1111/j.1369-7625.2004.00296.x

Levin, L., Gewirtz, S., &Cribb, A. (2017). Shared Decision Making in Israeli Social Services: Social Workers’ Perspectives on Policy Making and Implementation. *British Journal of Social Work*, *47*(2), 507–523. https://doi.org/10.1093/bjsw/bcw024

Louwers, A., Warnink-Kavelaars, J., Obdeijn, M., Kreulen, M., Nollet, F., &Beelen, A. (2018). Effects of Upper-Extremity Surgery on Manual Performance of Children and Adolescents with Cerebral Palsy: A Multidisciplinary Approach Using Shared Decision-Making. *The Journal of Bone and Joint Surgery*, *100*(16), 1416–1422. https://doi.org/10.2106/JBJS.17.01382

M.D., F., R.C., F., &V., R. (2000). Creating a healing environment: The importance of the service setting in the new consumer-oriented healthcare system. *Journal of Healthcare Management*, *45*(2), 91–107. Retrieved from http://ovidsp.ovid.com/ovidweb.cgi?T=JS&PAGE=reference&D=emed7&NEWS=N&AN=30143706

Mazurenko, O., &Hearld, L. R. (2015). Environmental factors associated with physician’s engagement in communication activities. *Health Care Management Review*, *40*(1), 79–89. https://doi.org/https://dx.doi.org/10.1097/HMR.0000000000000003

O’Connor, A. M., Graham, I. D., Visser, A., A.M., O., &I.D., G. (2005). Implementing shared decision making in diverse health care systems: The role of patient decision aids. *Patient Education and Counseling*, *57*(3), 247–249. https://doi.org/http://dx.doi.org/10.1016/j.pec.2005.04.010

Parikh, P., Pockaj, B., Wasif, N., Halyard, M., Wong, W., Kosiorek, H., …Gray, R. (2015). Multidisciplinary Shared Decision Making in the Management of Ductal Carcinoma In Situ of the Breast. *Annals of Surgical Oncology*, *22*(3), 516–521. https://doi.org/10.1245/s10434-015-4607-z

Peek, M. E., Gorawara-Bhat, R., Quinn, M. T., Odoms-Young, A., Wilson, S. C., &Chin, M. H. (2013). Patient Trust in Physicians and Shared Decision-Making Among African-Americans With Diabetes. *Health Communication*, *28*(6), 616–623. https://doi.org/10.1080/10410236.2012.710873

Pollard, S., Bansback, N., &Bryan, S. (2015). Physician attitudes toward shared decision making: A systematic review. *Patient Education and Counseling*, *98*(9), 1046–1057. https://doi.org/10.1016/j.pec.2015.05.004

Quinn, T. D., Wolczynski, P., Sroka, R., &Urman, R. D. (2018). Creating a Pathway for Multidisciplinary Shared Decision-Making to Improve Communication During Preoperative Assessment. *Anesthesiology Clinics*, *36*(4), 653. https://doi.org/10.1016/j.anclin.2018.07.011

Sharko, M., Wilcox, L., Hong, M. K., &Ancker, J. S. (2018). Variability in adolescent portal privacy features: how the unique privacy needs of the adolescent patient create a complex decision-making process. *Journal of the American Medical Informatics Association*, *25*(8), 1008–1017. https://doi.org/10.1093/jamia/ocy042

Siegel Corey, A. (2007). Embracing the internet for progress in shared decision-making. *Inflammatory Bowel Diseases*, *13*(12), 1579–1580. https://doi.org/10.1002/ibd.20259

Simmons, L. A., Wolever, R. Q., Bechard, E. M., &Snyderman, R. (2014). Patient engagement as a risk factor in personalized health care: A systematic review of the literature on chronic disease. *Genome Medicine*, *6*(2), 1–13. https://doi.org/10.1186/gm533

van derWeijden, T., vanVeenendaal, H., Drenthen, T., Versluijs, M., Stalmeier, P., Koelewijn-van Loon, M., …Timmermans, D. (2011). Shared decision making in the Netherlands, is the time ripe for nationwide, structural implementation?. *Zeitschrift Fur Evidenz, Fortbildung Und Qualitat Im Gesundheitswesen*, *105*(4), 283–288. https://doi.org/https://dx.doi.org/10.1016/j.zefq.2011.04.005

Visser, M., Deliens, L., &Houttekier, D. (2014). Physician-related barriers to communication and patient- and family-centred decision-making towards the end of life in intensive care: a systematic review. *Critical Care (London, England)*, *18*(6), 604. https://doi.org/https://dx.doi.org/10.1186/s13054-014-0604-z

Westermann, G. M. A., Verheij, F., Winkens, B., Verhulst, F. C., &VanOort, F. V. A. (2013). Structured shared decision-making using dialogue and visualization: A randomized controlled trial. *Patient Education and Counseling*, *90*(1), 74–81. https://doi.org/10.1016/j.pec.2012.09.014
